# Imeglimin in Patients with an Estimated Glomerular Filtration Rate Less than 45 mL/min per 1.73 m^2^: A Case Series

**DOI:** 10.31662/jmaj.2025-0169

**Published:** 2025-08-29

**Authors:** Shohei Fukunaga, Kei Matsumoto, Yudo Tanno, Takashi Yokoo

**Affiliations:** 1 Division of Nephrology and Hypertension, Department of Internal Medicine, The Jikei University School of Medicine, Tokyo, Japan

**Keywords:** chronic kidney disease, diabetes mellitus, Imeglimin, side effects

## Abstract

**Introduction:**

Imeglimin is a new oral antidiabetic agent that became available in Japan in 2021. It is not recommended for patients with an estimated glomerular filtration rate (eGFR) <45 mL/min per 1.73 m^2^ because of the lack of data regarding such patients. However, imeglimin may be beneficial for patients with chronic kidney disease because basic studies have shown its ability to decrease albuminuria and suppress kidney fibrosis.

**Methods:**

We performed a retrospective study of the safety of imeglimin for patients with an eGFR <45 mL/min per 1.73 m^2^. Side effects, renal function, liver function, pH, hydrogen carbonate, carbon dioxide, and glycated hemoglobin A1c when imeglimin was initiated and 3 months after treatment initiation were compared. Fifteen patients (10 male and five female patients) who were newly prescribed imeglimin treatment between October 1, 2021, and April 30, 2024, and had an eGFR <45 mL/min per 1.73 m^2^ at that time were enrolled in this study. However, one patient self-interrupted treatment because of lightheadedness; therefore, 14 patients (10 male and four female patients) were included in the analysis.

**Results:**

No deterioration in renal and hepatic functions occurred. Proteinuria decreased significantly, pH increased significantly, hydrogen carbonate remained unchanged, and carbon dioxide showed a decreasing trend. Subjective symptoms such as hypoglycemia and gastrointestinal symptoms were not observed.

**Conclusions:**

Short-term imeglimin treatment may be safe for patients with an eGFR <45 mL/min per 1.73 m^2^. Further studies of the safety of long-term imeglimin use are warranted.

## Introduction

Imeglimin is an oral antidiabetic agent that became available in Japan in 2021. It is the first drug in a novel class of oral antidiabetic agents known as glimins ^(1)^. Imeglimin targets mitochondrial function and improves insulin secretion and sensitivity ^(2)^. Although imeglimin and metformin have similar structures, imeglimin is associated with a low risk of lactic acidosis ^(3)^. Additionally, it promotes insulin secretion ^(4)^, protects pancreatic beta cells ^(5)^, improves insulin resistance in muscles and the liver ^(6)^, and inhibits gluconeogenesis ^(7)^. Furthermore, fibrosis suppression in the kidney has been observed ^(8)^, suggesting that imeglimin may be effective for the treatment of patients with chronic kidney disease (CKD). However, real-world data regarding imeglimin are lacking, and no reports of its safety for patients with advanced CKD are available. In addition, limited antidiabetic agents for the treatment of patients with CKD are available; therefore, more antidiabetic agents are needed. Currently, imeglimin is cautiously administered to patients with an estimated glomerular filtration rate (eGFR) <45 mL/min per 1.73 m^2^ because limited studies of its use for such patients exist. Therefore, the present study aimed to investigate the safety of imeglimin for patients with an eGFR <45 mL/min per 1.73 m^2^.

## Materials and Methods

### Study setting

This retrospective study was conducted at Jikei University Hospital and Katsushika Medical Center in Japan in accordance with the principles of the Declaration of Helsinki, and it was approved by the Institutional Ethics Committee [number 36-259(12371)] before the start of the study. We applied the opt-out method to obtain consent from the study participants using instructions posted on the website.

### Study population

This study included patients with an eGFR <45 mL/min per 1.73 m^2^ who initiated imeglimin treatment at Jikei University Hospital or Katsushika Medical Center between October 1, 2021, and April 30, 2024.

### Data collection

Age, sex, blood test results, imeglimin dosage, concomitant use of antidiabetic medications, and subjective symptoms were collected from the medical records and retrospectively reviewed. In addition, electronic medical records were searched to collect information regarding glycated hemoglobin A1c (HbA1c), eGFR, proteinuria, pH, hydrogen carbonate (HCO_3_^-^), carbon dioxide (CO_2_), aspartate aminotransferase, alanine aminotransferase, triglyceride, high-density lipoprotein cholesterol, and low-density lipoprotein cholesterol. To calculate the annual change in eGFR (eGFR slope), a linear approximation was applied to eGFR measurements obtained during the period 3 months before and 3 months after imeglimin treatment, and the slope of this line was used.

### Safety measurements

The adverse effects of imeglimin include metabolic acidosis, hypoglycemia, nausea, diarrhea, and constipation. The pH, HCO_3_^-^, and CO_2_ values of patients with metabolic acidosis were compared at the initiation of imeglimin treatment. Information regarding hypoglycemia, nausea, diarrhea, and constipation was collected from the medical records.

### Statistical analysis

Because this was a retrospective study, no statistical sample size calculations were conducted. The Wilcoxon signed-rank test was used for the nonparametric paired data analysis, and p<0.05 was considered statistically significant. Statistical analyses were performed using Prism version 7.0 (GraphPad Software Inc., San Diego, CA, USA). Data were presented as the mean ± standard deviation.

## Results

### Patient characteristics

Fifteen patients (10 male and five female patients) with an eGFR <45 mL/min per 1.73 m^2^ initiated imeglimin treatment between October 1, 2021, and April 30, 2024 (Table 1). However, 14 patients (10 male and four female patients) were included in the analysis because one patient self-discontinued treatment due to lightheadedness. The mean age of the patients was 67.9 years (±7.1 years). The mean duration of diabetes was 131 months (±53.7 months). The mean renal function was 28.7 mL/min/1.73 m^2^ (±13 mL/min per 1.73 m^2^). The mean proteinuria level was 2.01 g/gCr (±1.66 g/gCr) (one patient had nephrotic levels of proteinuria). The mean HbA1c level was 7.4% (±0.9%). Additionally, the mean imeglimin starting volume was 714.3 mg/day (±425.8 mg/day). Treatment was switched from other agents to imeglimin for eight patients. Switching from metformin to imeglimin was the most frequently performed treatment alteration (five patients), followed by switching from repaglinide (one patient), teneligliptin (one patient), and voglibose (one patient) to imeglimin. Furthermore, Sodium-Glucose cotransporter 2 (SGLT2) inhibitors were the most commonly used concomitant medications (seven patients).

**Table 1.** Patient characteristics at baseline.

Sex	
Male	10 (71.4%)
Female	4 (28.6%)
Age (years) (Mean ± SD)	67.9 ± 7.1
Duration of diabetes (months) (Mean ± SD)	131 ± 53.7
eGFR (mL/min/1.73m^2^) (Mean ± SD)	28.7 ± 13.0
Proteinuria (g/gCr) (Mean ± SD)	2.01 ± 1.66
HCO_3_^-^ (mmol/L) (Mean ± SD)	24.0 ± 4.4
HbA1c (%) (Mean ± SD)	7.4 ± 0.9
Dose of imeglimin
500 mg/day	10 (71.4%)
1000 mg/day	3 (21.4%)
2000 mg/day	1 (7.1%)
Switching from other medications	8 (57.1%)
Concomitant medications
SGLT2i	7 (50.0%)
GLP1	5 (35.7%)
DPP4i	4 (28.6%)
SU	2 (14.3%)
α-GI	2 (14.3%)
Insulin	2 (14.3%)
GLP1	1 (7.1%)
TZD	1 (7.1%)

α-GI, α-glucosidase inhibitor; DPP4i, dipeptidyl peptidase-4 inhibitor; eGFR, estimated glomerular filtration rate; F, female; GL, glinide GLP1, glucagon-like peptide-1 receptor agonist; HbA1c, glycated hemoglobin A1c; HCO3-, hydrogen carbonate; M, male; SGLT2i, sodium glucose transporter 2 inhibitor; SU, stunned myocardium; TZD, thiazolidinedione.

### Treatment safety

**Figure 1.**
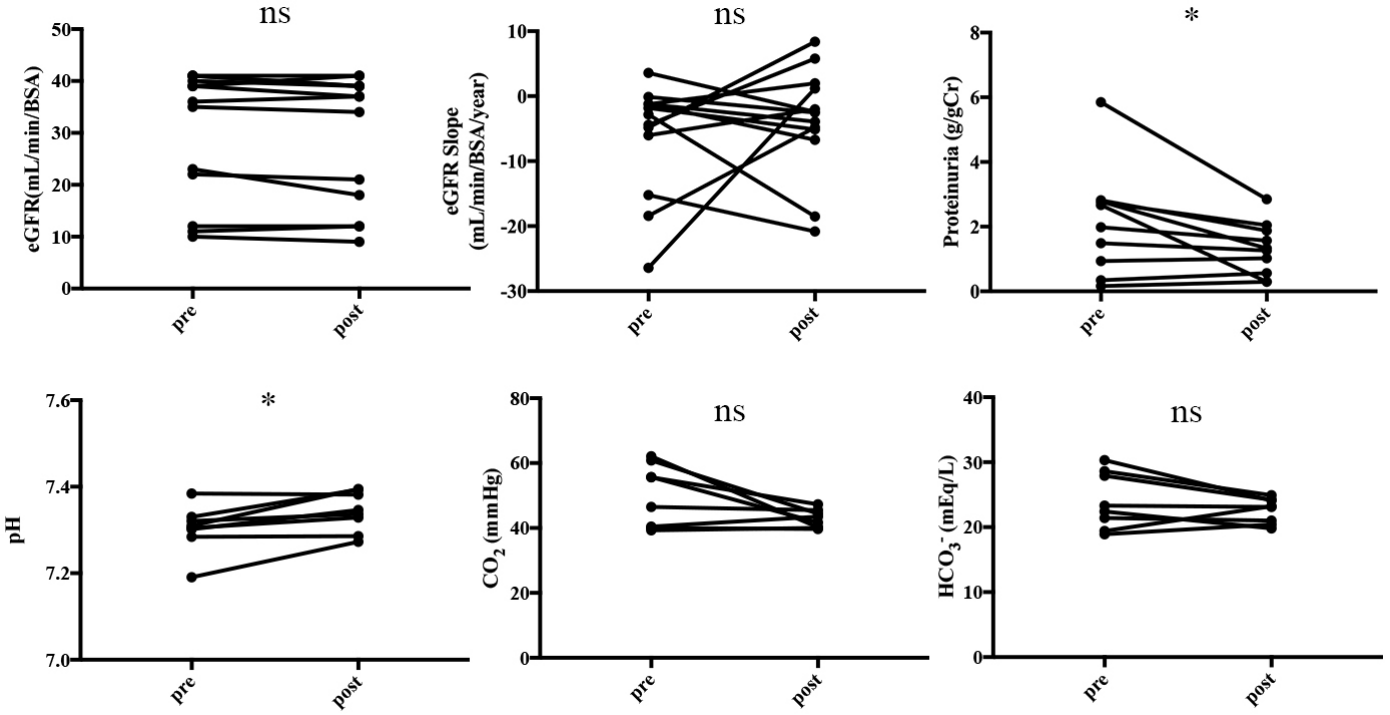
Comparisons of the eGFR, pH, CO_2_, and HCO_3_^-^ at the time of imeglimin initiation and 3 months after treatment initiation. The venous blood gas test revealed increased pH at the time of imeglimin initiation and 3 months after treatment initiation. At 3 months after treatment initiation, the eGFR, CO_2_, and HCO_3_^-^ were not significantly different, but CO_2_ exhibited a decreasing trend. *p < 0.05. Error bars in bar plots represent standard errors of the mean. CO_2_: carbon dioxide; eGFR: estimated glomerular filtration rate; HCO_3_^-^: hydrogen carbonate; ns: not significant.

The patient who discontinued imeglimin treatment because of lightheadedness did not experience hypoglycemia or worsening acidemia; therefore, the relationship between lightheadedness and imeglimin use was unclear. Other patients used imeglimin without adverse effects such as hypoglycemia, nausea, diarrhea, and constipation. Moreover, worsening of the renal function (eGFR and eGFR slope) was not observed after 3 months of imeglimin treatment. However, a significant decrease in proteinuria was observed (p = 0.027). Furthermore, venous blood gas tests were performed for eight patients. After imeglimin administration, the pH increased, resulting in a significant difference compared to that observed before treatment (p = 0.023). However, no significant difference in HCO_3_^-^ was observed. A decreasing trend (with no significant difference) in CO_2_ was observed (Figure 1).

### Treatment effects

The HbA1c level at treatment initiation was not significantly different from that at 3 months after initiation. In addition, the HbA1c level of patients who added imeglimin to their treatment regimen was not significantly different from that of patients who switched from other agents to imeglimin (7.6 ± 0.8 vs. 7.9 ± 1.0; p = 0.844). Moreover, liver enzymes (aspartate aminotransferase and alanine aminotransferase), triglycerides, and low-density lipoprotein cholesterol of the patients remained unchanged (Table 2).

**Table 2.** Comparison of Parameters at the Initiation of Imeglimin and at 3 Months after the Initiation of Imeglimin.

	Pre (Median ± SD)	Post (Median ± SD)	p
AST	24.5 ± 6.5	20.5 ± 4.2	0.073
ALT	18.0 ± 7.9	19.0 ± 6.3	0.318
TG	173.0 ± 70.0	175.0 ± 54.9	0.577
LDL	107.0 ± 30.5	110.0 ± 34.7	0.289
HDL	56.5 ± 17.0	51.0 ± 11.3	0.168
HbA1c (%)	7.40 ± 0.93	7.25 ± 0.99	0.866

ALT: alanine aminotransferase; AST: aspartate aminotransferase; HbA1c: glycated hemoglobin A1c; HDL-C: high-density lipoprotein cholesterol; LDL-C: low-density lipoprotein cholesterol; TG: triglyceride. Wilcoxon signed-rank test was used for data analyses, and p < 0.05 was considered statistically significant.

## Discussion

The number of patients with CKD is increasing worldwide ^(9)^, and the number of patients on dialysis is expected to increase 2.1-fold by 2030 compared with that in 2010 ^(10)^. Therefore, it is urgently necessary to control the new onset and progression of CKD. Diabetes mellitus causes CKD and plays a role in its progression. However, the use of several antidiabetic agents is reduced or discontinued as renal function deteriorates, thus limiting the use of antidiabetic agents in patients with CKD. Therefore, expanding the range of available antidiabetic agents for patients with CKD is warranted.

Imeglimin is the first in a new class of oral antidiabetic agents referred to as glimins for the treatment of type 2 diabetes. In a study of its efficacy and safety for Japanese patients with type 2 diabetes (TIMES1 study), imeglimin significantly reduced HbA1c compared to the placebo ^(11)^. The activity of imeglimin stimulates the secretion of glucose concentration-dependent insulin in pancreatic beta cells through its effects on the nicotinamide phosphoribosyltransferase gene and mitochondrial respiratory chain complex I ^(5)^. Additionally, imeglimin inhibits gluconeogenesis in the liver and skeletal muscles, improves glucose uptake ^(6)^, reduces excessive production of reactive oxygen species, and improves glucose homeostasis; these effects are likely related to its actions via mitochondria ^(7)^. Imeglimin and metformin have similar structural formulas. Specifically, imeglimin has the guanidino group found in metformin converted to a tetrahydrotriazine. Imeglimin administration is associated with a low risk of lactic acidosis according to animal ^(12)^ and human ^(3)^ studies.

Imeglimin ameliorated cardiac and renal damage in a diabetic model of Zucker fa/fa rats ^(8)^. Specifically, 90 days of imeglimin treatment reduced reactive oxygen species in left ventricular mitochondria and improved left ventricular function. Furthermore, albuminuria and fibrosis of the renal interstitium were reduced. Imeglimin causes a sustained reduction in oxidative stress and an increase in nitric oxide bioavailability, which may lead to renal protection. This report marks the first observation of urinary proteinuria reduction with imeglimin in humans. However, because of the limited scale and short duration of this study, and the unclear underlying mechanism of the effect of imeglimin on proteinuria, definitive establishment of its efficacy in humans based solely on these findings is challenging. Imeglimin is a potentially useful agent for patients with CKD, but its safety for patients with an eGFR <45 mL/min per 1.73 m^2^ has not been investigated; therefore, although its administration is not contraindicated, it should be administered with caution. In future studies of the efficacy of imeglimin for patients with CKD, it will be essential to establish its safety for patients with an eGFR <45 mL/min per 1.73 m^2^. However, real-world data of imeglimin for patients with CKD are limited. Only one study has evaluated its use for patients undergoing renal replacement therapy, and its sample size was small (six patients) ^(13)^. In that study, all six patients had type 2 diabetes, were on hemodialysis or peritoneal dialysis, and were treated with imeglimin 500 mg/day. The observation period was approximately 3 months; during that time, although no statistically significant difference in the HbA1c level was observed, a decreasing trend was evident. Imeglimin is a relatively well-tolerated agent with no specific adverse events. However, because imeglimin is primarily excreted by the kidneys, patients with impaired renal function experience an increased maximum concentration and area under the receiver-operator characteristic curve compared to those of patients with normal renal function ^(14)^; therefore, an increased number of adverse effects is possible.

In the present study, imeglimin was administered to patients with an eGFR <45 mL/min per 1.73 m^2^, and serious side effects such as gastrointestinal symptoms and hypoglycemic attacks were not observed. Lightheadedness was observed in one patient. Reports of imeglimin-related lightheadedness are not available; however, hypoglycemia can cause lightheadedness. Nevertheless, hypoglycemia was not observed in this case, and the causal relationship between imeglimin and lightheadedness was unclear. Decreased HCO_3_^-^ and worsening acidemia were not observed; however, the pH of patients was significantly increased. This increase in pH may be attributable to the decreasing trend of CO_2_. However, no basic or clinical studies have reported a relationship between imeglimin and CO_2_. This study was limited by its small sample size and retrospective design; therefore, a causal link between imeglimin and CO_2_ could not be established. Because other renal and hepatic dysfunctions were not observed, imeglimin could be safe for patients with an eGFR <45 mL/min per 1.73 m^2^. Therefore, further investigations are necessary.

This study had some limitations. Only two centers were included, and the sample size was small; consequently, studies with larger sample sizes are required. Furthermore, this study did not examine the dosage of imeglimin for patients with an eGFR <45 mL/min per 1.73 m^2^. Eight of the 14 patients switched from other diabetes medications to imeglimin, and worsening HbA1c levels were not observed. However, six patients who added imeglimin to their treatment regimen did not experience reduced HbA1c levels. The usual dosage of imeglimin for patients with an eGFR >45 mL/min per 1.73 m^2^ is 1,000 mg twice daily (2,000 mg/day). In one study that examined the dosage of imeglimin, no reduction in the HbA1c level was associated with the administration of imeglimin 500 mg twice daily (1,000 mg/day) as a monotherapy compared to placebo ^(15)^. Moreover, that study investigated patients with CKD stages G1, G2, and G3a and found that 500 mg twice daily (1,000 mg/day) was inadequate at all stages. The recommended dosage for patients with an eGFR of 15 to 45 mL/min per 1.73 m^2^ is approximately 500 mg twice daily (1,000 mg/day) based on population pharmacokinetics and dosage adjustment predictions analyses ^(16)^. This study had a retrospective design, and the dosage of imeglimin for patients with an eGFR <45 mL/min per 1.73 m^2^ was determined according to the discretion of each physician. Furthermore, in this study, the initial dose was 714.3 mg/day (±425.8 mg/day), which is less than the typical dosage; therefore, it may have been insufficient. We did not investigate the appropriate imeglimin dosage for patients with renal impairment; therefore, further studies should aim to determine the appropriate dosage for such patients.

Imeglimin treatment did not result in major complications or decreased renal function and HCO_3_^-^, when administered to patients with an eGFR <45 mL/min per 1.73 m^2^; therefore, its short-term use may be safe for these patients. However, because of the small sample size and short duration of this study, the complete safety of imeglimin could not be ensured. Furthermore, this study did not investigate the appropriate dosage of imeglimin; therefore, further prospective studies should be performed to confirm these results. Diabetes medications for patients with advanced CKD are limited. We hope that imeglimin will become available for patients with advanced CKD because its acceptance could ultimately lead to an increased number of available medications for such patients.

## Article Information

### Acknowledgments

We would like to thank Editage (www.editage.com) for English language editing.

### Author Contributions

Shohei Fukunaga, Kei Matsumoto, Yudo Tanno, and Takashi Yokoo

All authors contributed to the study conception and design. Material preparation, data collection, and analysis were performed by Shohei Fukunaga and Kei Matsumoto. The first draft of the manuscript was written by Shohei Fukunaga, and all authors commented on previous versions of the manuscript. All authors read and approved the final manuscript.

### Conflicts of Interest

None

### IRB Approval Code and Name of the Institution

No. 36-259(12371), the Jikei University School of Medicine.

### Informed Consent

Informed consent was not required for this study because of the use of anonymized data.
